# Operational demonstration and process evaluation of non-pneumatic anti-shock garment (NASG) introduction to the public health system of Northern Province, Zambia

**DOI:** 10.1186/s12913-023-10294-0

**Published:** 2023-11-29

**Authors:** Naomi Medina-Jaudes, Andy E. Carmone, Margaret L. Prust, Lupenshyo Ngosa, Oluwaseun Aladesanmi, Morrison Zulu, Andrew Storey, Beauty Muntanga, Caren Chizuni, Angel Mwiche, Hilda Shakwelele, Aniset Kamanga

**Affiliations:** 1 Clinton Health Access Initiative, Inc, Boston, MA USA; 2 Clinton Health Access Initiative, Inc, Lusaka, Zambia; 3 Zambia Ministry of Health, Kasama, Northern Province Zambia; 4 Zambia Ministry of Health, Lusaka, Zambia

**Keywords:** NASG, Maternal mortality, Obstetric Hemorrhage, Zambia

## Abstract

**Background:**

A disproportionate burden of maternal deaths occurs in low- and middle-income countries (LMICs), and obstetric hemorrhage (OH) is a leading cause of excess mortality. In Zambia, most of maternal deaths are directly caused by OH. The Non-Pneumatic Anti-Shock Garment (NASG) is a first aid tool that uses compression to the abdomen and lower body to stop and reverse hypovolemic shock secondary to OH. We describe the process and experiences introducing the NASG into the Zambia public health system to encourage the development of national policies, clinical guidelines, and implementation plans that feature the NASG.

**Methods:**

We conducted an observational study of NASG introduction to 143 public health facilities in Northern Province, Zambia, organizing observations into the five dimensions of the RE-AIM evaluation framework: reach, effectiveness, adoption, implementation, and maintenance. The NASG was introduced in August 2019, and the introduction was evaluated for 18 months. Data on healthcare worker training and mentorship, cases where NASG was used, and NASG availability and use during the study period were collected and analyzed.

**Results:**

The NASG was successfully introduced and integrated into the Zambia public health system, and appropriately used by healthcare workers when responding to cases of OH. Sixteen months after NASG introduction, NASGs were available and functional at 99% of study sites and 88% reported ever using a NASG. Of the 68 cases of recorded OH where a NASG was applied, 66 were confirmed as clinically appropriate, and among cases where shock index (SI) could be calculated, 59% had SI ≥ 0.9. Feedback from healthcare providers revealed that 97% thought introducing the NASG was a good decision, and 92% felt confident in their ability to apply the NASG after initial training. The RE-AIM average for this study was 0.65, suggesting a public health impact that is not equivocal, and that NASG introduction had a positive population-based effect.

**Conclusions:**

A successful NASG demonstration took place over the course of 18 months in the existing health system of Northern Province, Zambia, suggesting that incorporation of NASG into the standard of care for obstetric emergency in the Zambia public sector is feasible and can be maintained without external support.

**Supplementary Information:**

The online version contains supplementary material available at 10.1186/s12913-023-10294-0.

## Background

A disproportionate burden of maternal deaths occurs in low- and middle-income countries (LMICs), and obstetric hemorrhage (OH) is a leading cause of this excess mortality [1]. In Zambia, maternal death remains a tremendous challenge to the public health system and society in general. In 2018, it was estimated that Zambia’s maternal mortality ratio (MMR) was 282 per 100,000, and the majority of the maternal deaths were directly caused by OH [2, 3]. A recent evaluation of maternal death reviews (MDRs) at a rural hospital in Zambia found that maternal deaths from all causes decreased over the course of five years, with the exception of deaths from postpartum hemorrhage, a key type of OH [4]. Case reviews showed that issues of accessibility, such as poor road conditions, as well as issues related to the health system, such as delayed referrals, as well as lack of stabilizing measures before referral are major contributors to this persistent problem. This underscores the urgency of identifying wide-reaching, systems-based solutions to address mortality from OH in Zambia.

The Non-Pneumatic Anti-Shock Garment (NASG) is a first aid tool that uses compression delivered to the abdomen and lower body through five segments of neoprene secured with Velcro to stop and reverse hypovolemic shock secondary to OH. Anyone can be trained to apply a NASG, making it ideal for even community-level use. Once applied, bleeding is curtailed and blood is shunted to vital organs, buying precious time to identify the cause of the OH and get access to the place, people, and things required to provide the treatment for it. The value that NASG adds centers on this life-saving time gained to deliver the right care to women in shock who, without a temporizing measure, may have only hours or even minutes to live.

Multiple studies have already demonstrated that NASG improves clinical outcomes. A cluster randomized trial in Zambia and Zimbabwe showed a 46% mortality reduction associated with NASG; however, it should be noted that the desired sample size for statistical power was not reached in this study [5]. A meta-analysis of observational studies showed a 48% mortality reduction among women who received the NASG, compared to the standard of care [6]. A process evaluation of the introduction of the NASG in public sector facilities in Zimbabwe showed healthcare workers were able to correctly use the garment after training and the public health system could afford to incorporate and maintain its use [7]. In cases of hypovolemic shock where a NASG was used, there were no maternal deaths and no extreme adverse outcomes, however, because the implementation period used in this evaluation was not of sufficient duration to estimate public health impact and was carried out in one country, a similar process evaluation of a longer period in a different geography enhances value of the literature on incorporation of NASG in LMIC maternity services. The cost-effectiveness of NASG has also been demonstrated in several studies, including one in Zambia which found that the cost-effectiveness of early application of the NASG at primary health care level, compared to waiting until arrival at the referral hospital, was $21.78 per disability-adjusted life year (DALY) [8, 9].

In 2012, the World Health Organization (WHO) recommended NASG as a temporizing measure for women experiencing hypovolemic shock secondary to OH but noted that more information about its use at primary care level was needed [10]. Despite positive clinical evidence, demonstration of cost-effectiveness, and normative guidance recommending it, adoption of NASG into the standard of maternity care has not been rapid in many countries where the burden of maternal mortality is highest and most inequitable. To date, operational guidelines for rolling out NASGs are lacking and documentation of implementation experiences is scant.

Generally in LMICs and specifically in Zambia, there is an urgent need to improve early recognition of OH, stabilization, and access to definitive treatment. Introduction of NASG into the current standard of maternity care in LMICs represents an important opportunity to gain traction in the response to high maternal mortality from OH that has been elusive otherwise. Previous evidence on NASG use in Zambia comes from clinical studies in peri-urban areas with robust logistical and financial support. In order to encourage development of national policies, clinical guidelines, and implementation plans that feature NASG as an important part of an enhanced response to high rates of death from OH, experiences of using NASG in the public health system should be shared.

## Methods

The primary aim of this study was to identify critical reach, effectiveness, adoption, implementation and maintenance factors influencing NASG uptake in the obstetric emergency response system in public sector health facilities Northern Province, Zambia. Of particular concern was identifying and documenting best practices and learnings related to NASG clinical training and mentoring, training of cleaning staff, communication, transportation, and standard operating procedures related to referral so as to inform national policy and roll-out of NASGs in Zambia.

### Design

We conducted an observational study of NASG introduction to Northern Province, organizing observations into the five dimensions of the implementation research framework, RE-AIM: reach, effectiveness, adoption, implementation, and maintenance. The RE-AIM framework was originally designed to facilitate the translation of novel interventions to implementation; it was for this reason it was selected to facilitate evaluating the process of introducing this new first aid tool into the standard obstetric emergency response [11]. Within the RE-AIM framework, we adapted the definition of each dimension and outcome measure as laid out in Table 1.

**Table 1:** RE-AIM Framework Application to NASG Introduction in Zambia

Dimension	RE-AIM Definition	Outcome Measure
Reach	Measurement of participation; the absolute number, proportion, and representativeness of individuals who participate in a given initiative	• Proportion of program facilities with a provider well-trained in NASGs and prepared to use NASG 12 months after introduction • Proportion of program facilities that have applied a NASG 16 months after introduction
Effectiveness	Consequences of the initiative, which can be understood in terms of biologic indicators, whether positive or negative, and not excluding quality of life and economic outcomes	• Proportion of NASG applications that were clinically appropriate, within 6 months of introduction • Proportion of clients that had a NASG applied within 6 months of introduction
Adoption	The absolute number, proportion, and representativeness of settings and individuals who are willing to initiate a program	• Proportion of providers that believe introducing NASG was a good decision 6 months after introduction • Proportion of providers that reported feeling confident in using NASG 6 months after training
Implementation	The extent to which a program is delivered as intended	• Average total score on pre-training EmONC knowledge assessment for clinical staff taken as a baseline assessment • Average total score on post-training NASG knowledge assessment for clinical staff • Average total score on post-training NASG cleaning assessment for cleaning staff • Proportion of staff that received a passing score of 70% or higher on each assessment
Maintenance	The extent to which a program or policy becomes routine and part of the everyday norms and culture of the implementing entity	• Proportion of program facilities that had NASGs available and functional 16 months after introduction • Proportion of program facilities that reported no challenges with maintaining NASGs 16 months after introduction • Proportion of program facilities that reported no failure in NASG rotation scheme

The study was approved by the University of Zambia Biomedical Research Ethics Committee (reference number 126–2019). Further research clearance was also obtained from the National Health Research Authority (NHRA) as well as the relevant authorities from Ministry of Health officials at headquarters in the districts and from the facilities where data collection took place and reinforced through review by CHAI’s internal Scientific and Ethical Review Committee.

### Setting

**Fig. 1:**
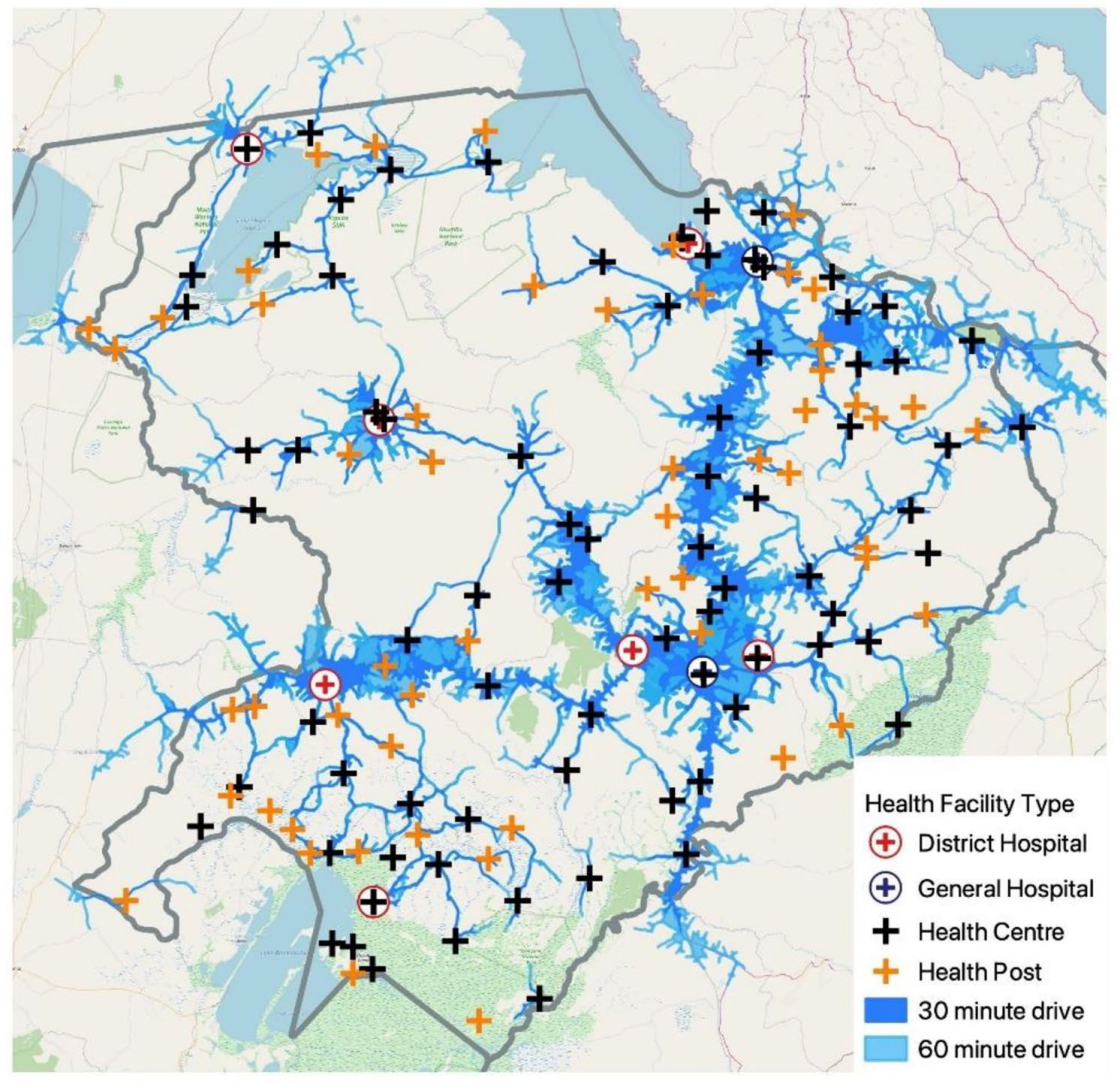
Distribution of and travel time to program-supported health facilities in Northern Province

The NASG was introduced in Northern Province as part of a broader Sexual, Reproductive, Maternal and Newborn Health (SRMNH) program implemented by the Zambia Ministry of Health (MOH) and the Clinton Health Access Initiative (CHAI) from 2018 to 2020. Within Northern Province, the program supported 143 public facilities serving approximately 1.5 million people and including 9 hospitals, 79 health centers, and 55 health posts. All health centers provide basic emergency obstetric and newborn care (BEmONC) while only hospitals provide comprehensive emergency obstetric and newborn care (CEmONC) services, including definitive treatment for hypovolemic shock from OH. In 2018, the year before NASGs were introduced, there were 41,301 live births, 374 cases of OH, and 45 maternal deaths reported to HMIS in the study geography. The map shown in Fig. 1 shows the distribution of program-supported facilities across Northern Province by facility type and with estimated travel times from the community to the nearest facility.

### NASG introduction

NASG was introduced to 143 program facilities in Northern Province beginning in August 2019. In partnership with the Provincial Health Office, CHAI made the catalytic procurement of NASGs and facilitated their distribution to the study facilities. It was determined that general hospitals would receive 20 NASGs, district hospitals would receive 10 NASGs, zonal health facilities would receive 7 NASGs, health centers would receive 5 NASGs, and health posts would receive 2 NASGs each.

In the same month that the NASG was introduced, mentors and health workers were trained on NASG use and completed a NASG skills assessment, which was integrated into existing EmONC training. Medical doctors, medical licentiates, clinical officers, nurses, midwives, community health assistants at supported facilities were eligible for training. Prior to the training, clinical staff were assessed on their EmONC knowledge including management of shock, patient assessment, bleeding after childbirth, management of the third stage of labor, and infection prevention. During training, clinical staff were trained in protocols for recognizing and addressing OH, indications for NASG use, NASG application and removal, integration of NASG into the obstetric emergency referral system, and garment cleaning and replacement logistics. Following training, clinical staff were assessed on their NASG knowledge including NASG purpose, application process, patient transfer, removal and safety, and cleaning staff were assessed on their knowledge of the care and cleaning of NASGs. The skills learned during training were then reinforced by mentorship visits that began the following month. NASG content was blended into pre-existing clinical mentorship programming, which was then utilized to follow up on adoption, implementation, and maintenance of NASGs across participating facilities.

From August 2019 to February 2020, all cases of OH at program facilities were meant to be tracked using an OH case form that documented the clinical assessment of the woman on presentation, whether a NASG was applied, whether transport to a referral facility was called and secured, any treatment provided, and clinical outcomes. The information collected on the OH forms, supplemented by case notes where necessary, was the chief source for assessing whether an application of the NASG was clinically appropriate, one of two outcome measures in the Effectiveness dimension. Clinicians on the study implementation team did a first review of NASG cases, and the assessments were repeated by another clinician. Any conflicts were resolved through discussion of available clinical data. In February 2020, about six months after NASG introduction, health workers that participated in the initial training were asked to participate in brief questionnaires on their perceptions of and confidence in using NASGs. From September to October 2020, NASG-focused mentorship visits were conducted in all program facilities, during which mentors used mentorship checklists to record the number of staff trained on NASGs at each facility and assess NASG use and maintenance in the facility, including availability of NASGs, application, removal and cleaning of NASGs, and NASG use in the most recent quarter. Of the 143 program facilities, 47 had documentation of all mentorship checklists from the study period and thus were considered for analysis for this study. In September and December 2020, program staff visited all program sites to collect basic data on NASG availability and use.

### Data collection and analysis

Data were initially captured in paper-based forms and then entered into an electronic database using SurveyCTO. Additional data about the number of births and cases of obstetric hemorrhage during the study period were extracted from the Zambia Health Information Management System (HMIS). Once collected and extracted, data were cleaned and analyzed using Stata 14 and Excel. Knowledge tests were analyzed against an answer key for each individual question, which generated scores for each section and for the full test. A score of 70% was pre-determined to be a passing score. Quantitative, structured data were analyzed using descriptive statistics. Any responses to open-ended questions were categorized thematically.

## Results

### Reach

**Fig. 2:**
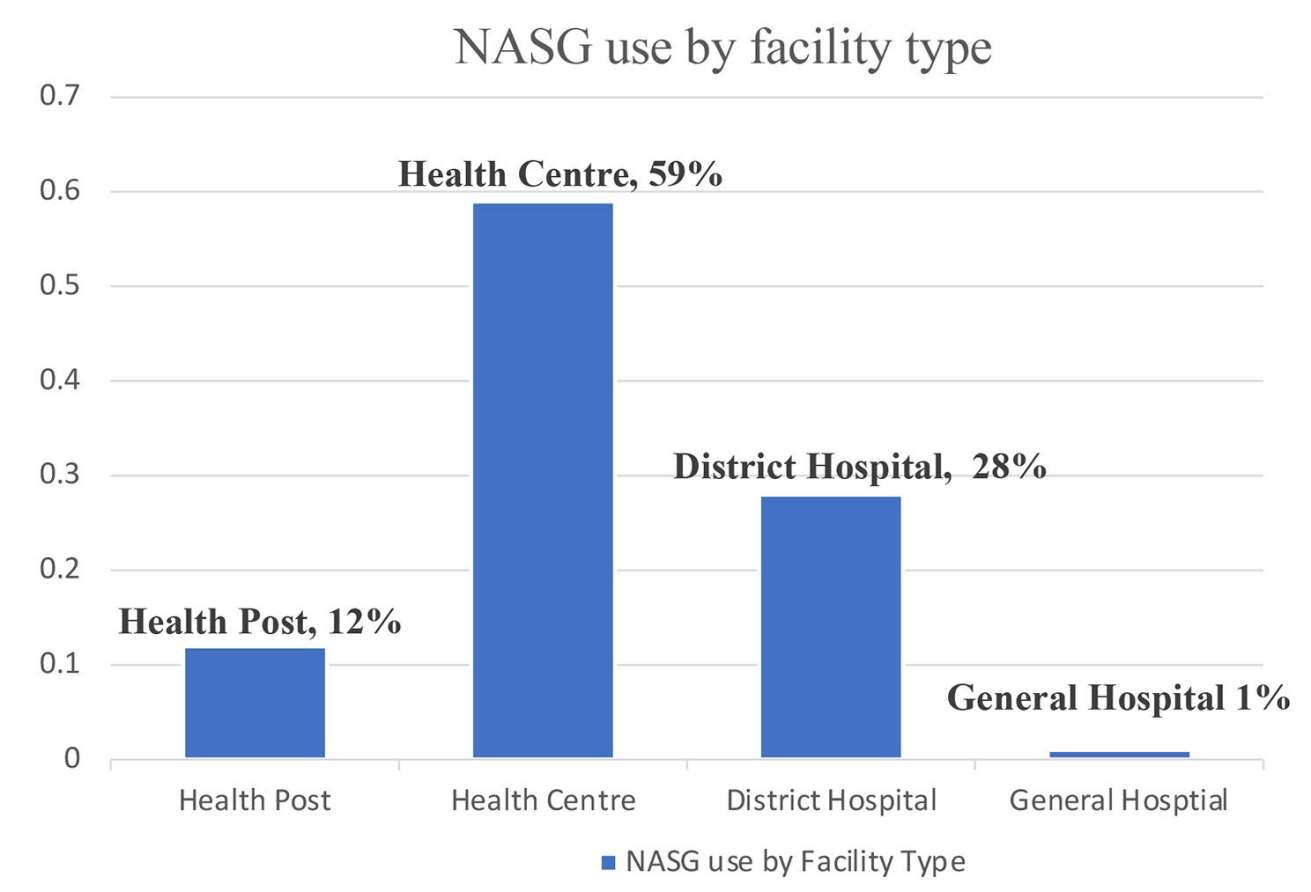
Proportion of cases where a NASG was applied, by facility type

Data from NASG-focused mentorship visits at 47 study sites revealed the extent to which NASG training and NASG use had been integrated into the emergency obstetric response network. Twelve months after NASGs had been introduced, 55% of the 47 study sites where data from NASG-focused mentorship visits were analyzed had at least two clinical staff trained on NASG use, with health centers and hospitals more likely to have at least two clinical staff trained compared to health posts (Table 2). Furthermore, NASGs were available at 96% of study sites where data from mentorship visits were analyzed and 57% of these sites reported applying a NASG in the last quarter; based on the facility assessments conducted at 16 months after NASG introduction, 88% of the 143 facilities had used NASG at least once (Table 2). Health posts had an average of two NASGs in stock per facility, while health centers and hospitals had an average of four and eight NASGs per facility, respectively. Figure 2 depicts the distribution of NASG uses during the study period by type of facility, illustrating the breadth of uptake of NASG in the health system. NASGs were primarily applied at health centers and health posts, which are primary care level facilities, and lack capacity to deliver the full suite of definitive treatment for OH.

### Effectiveness

From August 2019 to February 2020, 25,665 births and 365 cases of OH (1.4% of reported births) were reported to HMIS at the study sites. Of the 365 reported OH cases, 83 OH Case and Transfer Forms were completed and collected from the study sites. The 83 forms included the 68 NASG uses reported in the study period; 68 was greater than 55, or 15% of the 365 reported OH cases anticipated to progress to hypovolemic shock. Three quarters (52 of 68, 76%) of NASG uses were for shock secondary to postpartum hemorrhage (PPH), with post-abortion hemorrhage and antepartum hemorrhage equally represented as the next most common types of OH where NASG was applied. Among the 68 uses of NASG, two deaths occurred. In both cases, the women arrived at rural health centers conscious but in hypovolemic shock secondary to uncontrolled postpartum hemorrhage that began outside of the facility. A NASG was applied and then ambulances were secured to transport both women to referral facilities, but one woman died before she could be admitted to the hospital. The ambulance to transport the second woman was delayed due to poor road conditions and she experienced organ failure and death before receiving definitive treatment, despite receiving both oxytocin and misoprostol and having the NASG applied.

**Fig. 3:**
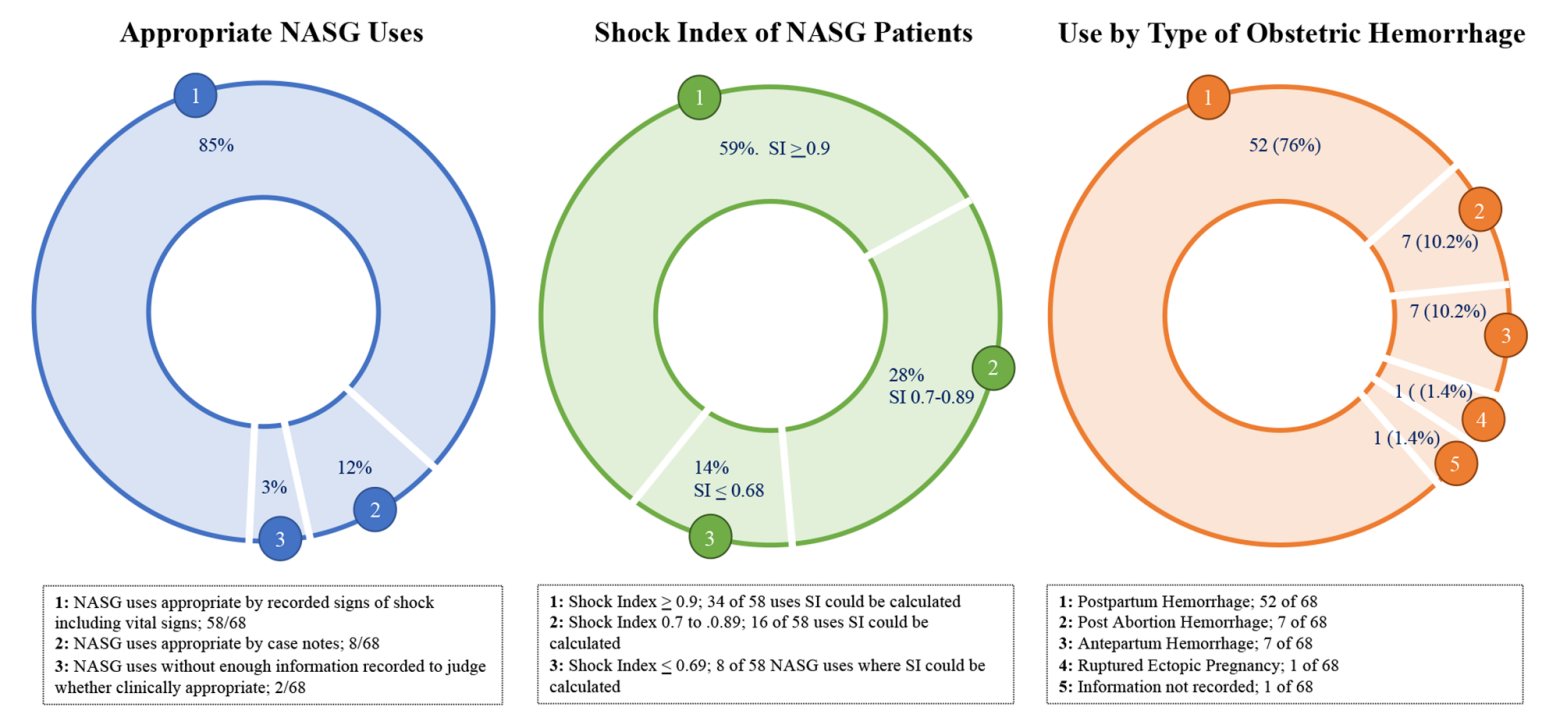
Characteristics of NASG uses: Whether clinically appropriate, the Shock Index (SI) of 58 of the 68 patients that had NASG applied, and Type of OH that precipitated need for NASG

Clinician study team members reviewed case notes of the 68 cases of OH in which a NASG was applied, confirming that 66 were clinically appropriate; 2 of the 68 cases did not have enough information recorded to verify (Table 2). 58 of the 68 NASG uses had enough information for the Shock Index (SI) to be calculated. SI, determined by dividing heart rate by systolic blood pressure, is a measure widely used in emergent situations to triage patients; a value ≥ 0.7 suggests urgent treatment is needed [12]. Research suggests that women with OH with SI ≥ 0.9 should be transferred immediately to the highest level of available care, and that SI > 0.9 predicts need for massive transfusion with 93.8% sensitivity. [13, 14] Among the cases where SI could be calculated, 59% ≥ 0.9, indicating the acute severity of the OH cases where NASG was used (Fig. 3).

### Adoption

Feedback from providers six months after NASG introduction revealed the extent to which the NASG had been accepted by facility staff as a tool for reducing maternal mortality from OH. Of the 211 providers who participated in the initial training and provided feedback, 97% of respondents indicated that introducing the NASG is a good decision and 94% reported that they think the NASG can help save lives by stabilizing patients until care can be delivered. Furthermore, 92% felt confident in using NASGs after the initial training, and this increased to 93% after receiving additional mentorship and support (Table 2). In open-ended questions, 28% of respondents to the feedback questionnaires included positive information about the NASG, noting that it is “very easy to use and very effective” and that “NASG training is so far the best initiative in trying to reduce the maternal death rate.” Respondents also included stories of cases where a NASG was used, writing that the “NASG is helping [save] lives” and that they “witnessed [a] patient at [a] District Hospital who survived after using NASG.” 3% of respondents voiced support for continued mentorship on NASGs as “[ongoing mentorship] will lead to perfection.”

### Implementation

Prior to the clinical training on the NASG for study site staff, 248 clinical staff were assessed on their EmONC knowledge based on a series of true and false questions. Four knowledge areas were assessed: management of shock and rapid initial assessment, bleeding after childbirth, management of third stage of labor, and infection prevention. Analysis of the pre-training tests revealed an average total score of 56%, with only 7% of participants receiving a score of 70% or higher. Participants scored lowest on the bleeding after childbirth section of the test (average score of 39%) (Table 2).

Following training on the NASG, 300 clinical staff were assessed on their NASG knowledge through a series of true and false questions about various aspects of NASG use. Six knowledge areas were assessed: NASG purpose, application, transfer/referral, procedures and referral, removal, and safety. Analysis of the knowledge test results revealed an average total score of 79%, with participants scoring lowest on the NASG removal section of the assessment (average score of 70%). 57% of participants received a total score of 70% or higher. 219 cleaning staff were also assessed after training on the care and cleaning of NASGs. The average total score for the post-training cleaning test was 79%, and 83% of participants received a score of 70% or higher (Table 2). 36% of respondents incorrectly answered a question about how long to soak the NASG in bleach solution: while the correct answer is 10 min, most participants chose 15 min, which can de-grade the neoprene of the NASG.

### Maintenance

Results from visits to all 143 program sites in the third and fourth quarter of 2020 demonstrated that the NASG was integrated into the standard of care with few challenges to maintenance. Sixteen months after the NASG was first introduced, 99% of study sites had NASGs available and functional. In the fourth quarter of 2020 at 16 months after introduction, 88% of study sites reported ever using a NASG, an increase from 80% in the third quarter. In that same period, NASG use increased most notably among health posts. Furthermore, nearly all facilities (93%) reported no challenges with maintaining NASGs for use in the second half of 2020 (Table 2). Of those that did report challenges, responses focused on the failure of the rotation scheme, or not receiving a clean NASG after referring a woman wearing one. However, at the time of the site visits, all except one facility had a NASG in stock, indicating that if a NASG was needed, nearly all facilities would have one available for use.

**Table 2:** Results from NASG Introduction in Northern Province

Outcome Measure	Result
**Reach** ^**1**^	
Proportion of program facilities with at least two trained providers at 12 months after NASG rollout	55% (26/47)
Proportion of program facilities that ever applied a NASG as of 16 months after introduction	88% (126/143)
**Effectiveness** ^**2**^	
Proportion of NASG applications that were clinically appropriate	97% (66/68)
Proportion of NASG clients documented to have recovered from hypovolemic shock	63% (43/68)
**Adoption** ^**3**^	
Proportion of providers that believe introducing NASGs is a good decision	97% (205/211)
Proportion of providers that feel confident in using NASGs after training	92% (194/211)
**Implementation** ^**4**^	
Average total score on post-training NASG knowledge assessment for clinical staff	79%
Proportion of staff that received a passing score of 70% or higher on post-training NASG knowledge assessment	57% (171/300)
Average total score on post-training NASG cleaning assessment for cleaning staff	79%
Proportion of staff that received a passing score of 70% or higher	83% (182/219)
**Maintenance** ^**5**^	
Proportion of program facilities that had NASGs available and functional 16 months after introduction	99% (142/143)
Proportion of program facilities that reported no challenges with maintaining NASGs 16 months after introduction	97% (131/141)^6^
Proportion of program facilities that reported no failure in NASG rotation scheme	93% (131/141)^6^

^1^The reach data point on trained providers comes from the mentorship checklist data, which were collected in September and October 2020. The reach data point on application of NASGs comes from the facility assessment data, which were collected in December 2020.

^2^All effectiveness data points are from the OH case and transfer data. Data were tracked and collected from August 2019 through February 2020.

^3^All adoption data points are from health worker feedback questionnaire data. Data were collected in February 2020.

^4^All implementation data points are from skills and knowledge assessment data. Data were collected in August 2019.

^5^All maintenance data points are from facility assessment data. Data were collected in December 2020.

^6^Two facilities did not provide a response to the question on challenges.

### RE-AIM Profile and Estimated Public Health Impact

**Fig. 4:**
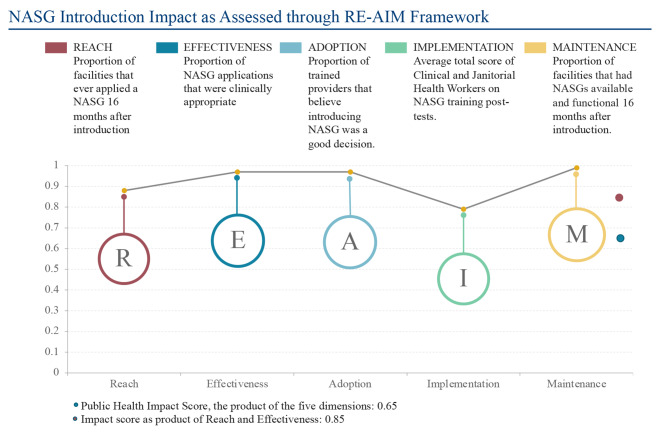
Assessment of NASG Introduction through RE-AIM Dimensions

The RE-AIM profile and average, shown in Fig. 4, plots one key assessment for each of the five dimensions as well as the public health impact score and an impact score of NASG introduction in our study setting [15]. The profile provides a quick visual expression of the strongest aspects of the intervention. The RE-AIM average, on the other hand, provides a single sum characterization of the process; the product of the five dimensions is the public health impact score, also understood as the population-based effect of the intervention [16]. The impact score, on the other hand, is the product of Reach and Effectiveness [17]. In our application of the RE-AIM framework, there were multiple outcome measures in each dimension. To calculate the impact scores, the most relevant measure for each dimension was utilized, with the exception of the Implementation dimension, where two outcome measures were represented as one. They happened to be of the same value, so no average of the two was necessary.

In Reach, proportion of participating facilities that used NASG during the study (88%) was included. In Effectiveness, proportion of clinically appropriate NASG uses (97%) was selected. For the Adoption dimension, proportion of providers reporting support for the decision to introduce NASG (97%) was included, while for Implementation, the average score on post-training tests for both clinical and cleaning staff (79%) was included. In Maintenance, the proportion of facilities where NASGs were observed to be functional and available (99%) was utilized in the calculation. The product of these five values is 0.65. This is to be interpreted with the understanding that the closer the score is to 1, the greater the public health impact of the intervention [16]. The score in this study was 0.65, suggesting a public health impact that is not equivocal, and that NASG introduction had a positive population-based effect. When the alternative impact calculation, the product of only Reach and Effectiveness is considered, the score is 0.85, which is higher, but excludes the crucial nuances of the broader process.

## Discussion

Prior to this work, the NASG was introduced in Zambia in two provinces (not including Northern Province) as part of a clinical trial, but its integration into the Zambian public health system has not fully been achieved, partly due to the lack of evidence related to the implementation process. The introduction of the NASG through this SRMNH program represents the first large-scale introduction outside of a controlled study setting in Zambia and demonstrates that the NASG can be implemented, adopted, and utilized by healthcare workers in public sector facilities successfully, including maintaining a sustainable rotation scheme without heavy outside support.

Results from this process evaluation revealed the importance of the NASG particularly when responding to cases of OH in the community or lower-level health facilities. Most of the cases where a NASG was applied involved referral to another facility, as opposed to utilizing NASG while preparing treatment at facilities where definitive treatment was available. Health centers and health posts, which are primary care level facilities, utilized the NASG most, indicating its usefulness when confronting maternal complications that occur closer to the community. In Northern Province, typical travel time from health posts and health centers to a referral hospital can range from two hours in the dry season to nearly six hours in the rainy season due to poor road conditions. Without the use of a NASG, these travel times may prove too long for a woman already in shock and whose condition is worsening rapidly from unabated bleeding.

Responding to OH as early as possible with stabilization and definitive treatment saves lives, but when definitive treatment is at a different location, stabilization during transport and while waiting for treatment to be delivered is paramount. NASG provides that stabilization, buying time, which is crucial to service delivery that is both client-centered and of good quality. By assuring stabilization during any transfer to a higher-level facility in the network, NASG supports networks of care where women can choose to deliver in facilities closer to where they live. Supporting client choice of birthing site through equipping networks of care with NASG, among other interventions, has been shown to improve health outcomes for pregnant women and newborns [18].

The success of the rotation scheme of the NASGs throughout the province largely relied on the emergency transport system in Zambia, allowing lower-level health facilities to be re-supplied with NASGs even if they referred women out wearing one. In 2018, as part of the release of the National Maternal and Neonatal Referral guidelines, Zambia launched a national initiative to increase access to ambulance services. The intended purpose of the guidelines was to improve the health outcomes of maternal and newborn care and they outline the referral process, criteria, checklists, communication, transfer, and the maternal and neonatal referral forms which provides feedback from the recipient facility [19]. However, the guidelines have not highlighted a linkage between communities in the remote areas and health facilities. This clearly leaves the pregnant women from the rural communities to decide how they get to the facility and further delays the delivery of care and treatment. Despite this gap among the study facilities, access to emergency transport for referral of patients was widely available, and this transportation was utilized to carry out NASG rotation, providing referring facilities with a clean NASG when they sent a patient wrapped in one. This lack of logistical challenge for NASG rotation relative to experience reported from other countries is worth noting [7].

While the utilization and rotation of the NASG were successful, pre-training EmONC scores were lower than expected and suggest that NASG training sessions should include EmONC-refresher information. Even in a setting where EmONC-related mentorship and training was being provided, EmONC knowledge before NASG training was more limited than anticipated. Further, the reported incidence of OH both through HMIS as well as through study data collection, was extremely low. This underreporting of OH could be due to lack of recognition of OH, OH occurring outside of facilities where it cannot be recorded, simple lack of documentation, or a mixture of reasons. The large proportion of dangerously high SI values among NASG uses suggests that late recognition and response to OH may be significant factors in Northern Province.

Based on interviews from key informants in Ethiopia, India, Nigeria and Zimbabwe, the key barriers and facilitators to rolling out NASGs nationally were initial cost, lack of political will by the government to support the program, lack of clear plan to return and exchange the garment among facilities, challenge in getting to rural and remote areas, some resistance by healthcare workers and weak health systems [20]. Throughout our evaluation, we were aware of these reported barriers and attempted to mitigate them to ensure a successful introduction of NASGs in Northern Province to serve as evidence to underpin an MOH decision to scale-up NASG as an integral part of OH response. There were few reports of the NASG being out of stock at facilities, suggesting that a sufficient number of NASGs were distributed to facilities at the start of the study. Health posts and health centers should have a minimum of two and five NASGs available respectively, while more should be available at tertiary level facilities (10–20 for hospitals). To ensure that the NASG is available at all facility levels even when a woman is referred out wearing one, the NASG rotation scheme should be integrated into the emergency transport system. A minimum of two clinical staff at each facility should be trained on the NASG. Mentorship visits were key to reinforcing any skills and knowledge gained during initial training sessions and gave providers the confidence to utilize the NASG when presented with a case of OH. Mentorship visits should be conducted monthly to provide support and reinforcement to providers.

The success of the NASG introduction in Northern Province has driven the MOH to pursue action towards scaling up the introduction of NASGs nationwide. In the facilities where NASGs were introduced through this study, NASGs continue to be utilized in cases of obstetric hemorrhage, and other provinces have expressed interest in introducing NASGs. Furthermore, the Zambia MOH has indicated support for scaling up the introduction of NASGs across the country. In partnership with CHAI, a policy brief is currently being developed, based on findings and learnings from this process evaluation, which would help facilitate the scale-up of NASGs throughout Zambia. In preparation for this, most districts in Northern Province have incorporated funds into their budgets that would allow them to procure NASGs. CHAI made the initial procurement of NASGs but following the results of this demonstration, the Zambia MOH committed to procuring NASGs for facilities across the country. However, the emergence of the COVID-19 pandemic disrupted this progress as funds intended to be used to procure NASGs were deviated to bolster the COVID-19 pandemic response in Zambia. In 2022, the MOH announced that the NASG would be included in the annual MOH budget. Likewise, CHAI provided initial support for MOH mentors to travel to sites for mentorship visits, but the MOH has committed to providing funding for on-going mentorship.

### Limitations

This evaluation had several limitations to note. Documentation in OH Case and Transfer Forms was limited; the emergency nature of OH cases meant that information included on the OH Case and Transfer Forms was not always complete, leaving gaps in understanding the condition of each woman when the NASG was applied and as she was transferred to the appropriate facility. Furthermore, OH Case and Transfer Forms were meant to be filled in for every case of OH, regardless of NASG application, during the study period. Beyond those cases when NASG was applied, only some additional data collection forms for OH were filled in. As such, it was not possible to assess whether there were cases of hypovolemic shock when a NASG was not used but could have been. This mirrors broader OH underreporting. Additionally, while the same individuals participated in the clinical pre- and post-cleaning test, it was not possible to link results for individuals across the two tests as identifying information was not collected. Information on the cadre of test participants was also not collected and thus results could not be disaggregated based upon whether the participant was a nurse versus a medical officer, for example. The duration of this study satisfied the minimum time recommended by the authors of the RE-AIM framework to calculate a public health impact score, but the time frame suggested, 6 to 12 months, is arbitrary [11]. Due to time and resource limitations, the dimension of effectiveness in this study assessed the appropriate use of NASG, whereas the more salient point would have been whether NASG reduced maternal mortality.

## Conclusions

A successful NASG demonstration took place over the course of 18 months in the existing health system of Northern Province, Zambia, suggesting that incorporation of NASG into the standard of care for obstetric emergency in the Zambia public sector is feasible and can be maintained without external support. NASGs were readily used as a tool to address hypovolemic shock secondary to obstetric hemorrhage, health workers from a range of cadres demonstrated adequate knowledge after training on NASGs and provided positive feedback, and existing systems were sufficient to ensure maintenance of NASG supply. This work exposed the need for clinical refreshers in emergency obstetric care which should be considered in planned NASG training. In order to advance availability of the NASG as a life-saving intervention, more documentation of implementation experiences is needed, as well as operational guidelines that can be adapted to varying contexts.

### Electronic supplementary material

Below is the link to the electronic supplementary material.

Supplementary Material 1

Supplementary Material 2

Supplementary Material 3

Supplementary Material 4

Supplementary Material 5

Supplementary Material 6

Supplementary Material 7

## Data Availability

The quantitative datasets created and analyzed during this study are available as Supporting Information files.
